# Differences in the Gene Regulatory Network for Floral Induction in Two *Camellia* Species

**DOI:** 10.3390/ijms262210854

**Published:** 2025-11-08

**Authors:** Xiong Wang, Weixin Liu, Jiyuan Li, Hengfu Yin, Xinlei Li, Minyan Wang, Zhengqi Fan

**Affiliations:** 1 Research Institute of Subtropical Forestry, Chinese Academy of Forestry, Hangzhou 311400, China; wangxiongrickxy2@163.com (X.W.); lwx060624@163.com (W.L.); jiyuan_li@126.com (J.L.); hfyin@sibs.ac.cn (H.Y.); lixinlei2020@163.com (X.L.); w524270986@163.com (M.W.); 2 Zhejiang Key Laboratory of Forest Genetics and Breeding, Hangzhou 311400, China

**Keywords:** *Camellia azalea*, *Camellia japonica*, floral induction, flowering regulation

## Abstract

The formation of plant flower buds is regulated by various genes occurring upstream in floral induction pathways. However, the precise regulatory roles and underlying molecular mechanisms of these pathways in *Camellia* flower bud formation remain unclear. This study investigated the annual periodicity pattern of flower bud formation in two *Camellia* species exhibiting distinct flowering phenotypes: *Camellia azalea*, which initiates flower buds continuously throughout the year, and *Camellia japonica*, which forms buds only between May and July. *C. azalea* helps address the lack of summer-flowering representatives within the *Camellia* genus. Elucidating its unique molecular mechanism of flowering regulation provides valuable guidance for breeding new cultivars with summer blooming traits. Comparative transcriptome analysis of mature leaves sampled annually from the two *Camellia* species revealed the highest number of differentially expressed genes (DEGs) in *C. azalea* between May and December, whereas in *C. japonica*, the peak number of DEGs occurred between June and December. Gene ontology analysis indicated that the most enriched category in the transcriptomes of both species was oxidoreductase activity, which was followed by cofactor binding in *C. azalea*, whereas in *C. japonica*, it was cellular amino acid metabolic process. Flowering-related genes were identified from the transcriptome database, yielding 248 transcripts in *C. azalea* and 257 in *C. japonica*. The transcriptome analysis also revealed that *C. azalea* lacks certain floral inhibitory pathways that are present in *C. japonica*, such as the photoperiod pathway genes including *GI2, FKF1,* and *COL14* and the thermosensitive pathway gene *SVP*. The reliability of the transcriptome results was further validated by quantitative real-time PCR (qRT-PCR) analysis. These results suggest that differences in upstream regulatory mechanisms within the floral induction pathways of *C. azalea* and *C. japonica* may underlie the species-specific patterns in the annual distribution of flower bud formation.

## 1. Introduction

Plant flowering is a complex process regulated by both endogenous and exogenous signals. Plants perceive a range of environmental cues to initiate floral induction and promote flower bud formation. Research has shown that the pathways of floral induction in plants include the photoperiodic, vernalization, gibberellin (GA), thermosensitive, age, and autonomous pathways [1,2,3]. These pathways involve a series of interconnected signaling pathways that respond to internal and external signals—such as light, temperature, and hormones—to regulate the expression of flowering-related genes, ultimately directing the shoot apical meristem to transition into a floral meristem and initiate flower bud development [4,5,6]. Different plant species exhibit distinct annual patterns of flower bud formation, which is a major contributor to the variation in flowering phenotypes among plants.

In the photoperiod pathway, *GIGANTEA* (*GI*) is an enhancer of *CONSTANS* (*CO*)*,* which can bind with *FLAVINBINDING KELCH REPEAT F*-*BOX1* (*FKF1*) to activate *CO* expression through the CYCLING DOF FACTOR1 (CDF1) protein [7,8]. A ZmLUX2-ZmFKF1a–ZmGI1-ZMM4 regulatory module is found that fine-tunes photoperiodic flowering of day-neutral temperate maize lines [9]. *BRASSINAZOLE RESISTANT 1* (*BZR1*), a key transcription factor in the phytohormone brassinosteroid (BR) signaling pathway, directly binds to the proximal promoter region of *CO* to suppress its transcription during long days, thus regulating photoperiodic flowering [10]. *GI* can function both as a promoter and a repressor of flowering, depending on the species and environmental context. In rice, overexpression of the *GI* homologous gene *OsGI* was reported to delay flowering [11]. In *Arabidopsis*, *CO* and 16 other *CONSTANS-LIKE* (*COL*) genes constitute the *CO* gene family [12,13]. Different members of this family exhibit divergent responses to the photoperiod: *COL1* and *COL2* are insensitive to photoperiodic changes [14], whereas *COL9* acts as a flowering repressor in *Arabidopsis* and *COL10* functions similarly in rice [15,16]. In *Chrysanthemum*, overexpression of a *COL16* homologous gene promoted early flowering in *Arabidopsis* [17]. In the vernalization pathway, the expression of the floral inhibitor gene *FLOWERING LOCUS C* (*FLC*) was inhibited after vernalization in *Arabidopsis*, leading to an increase in *FLOWERING LOCUS T* (*FT*) gene expression, thereby promoting flowering [18,19]. *GIBBERELLIN 3-OXIDASE* (*GA3OX*)*, GIBBERELLIN 20-OXIDASE* (*GA20OX*), and *GIBBERELLIN 2-OXIDASE* (*GA2OX*) are the main regulatory genes in the GA pathway. GA3OX and GA20OX can catalyze the biosynthesis of active GA, whereas GA2OX can inactivate them, thereby modulating the levels of active GA in the plant and ultimately influencing growth and development [20,21]. In the autonomous pathway, genes such as *FLOWERING TIME CONTROL LOCUS A* (*FCA*) and *FVE* can promote flowering by inhibiting expression of *FLC* [22,23]. In the age pathway, *miR156* acts as an inhibitory factor for flowering, whereas *miR172* is a promoter of flowering. During *Arabidopsis* development, the expression of miR156 gradually declines, while that of miR172 increases, and together they facilitate the transition to flowering [24,25]. In the thermosensitive pathway, a moderate rise in temperature induces the expression of both *FCA* and *PIF4*. *PIF4* directly binds to the *FT* promoter to activate transcription, while *FCA* further enhances *FT* expression, collectively promoting flowering [26,27]. Additionally, *SHORT VEGETATIVE PHASE* (*SVP*) encodes an important transcription factor in the thermosensitive pathway. Studies in kiwifruit and apple have shown that *SVP* represses flower bud formation by suppressing *FT* expression [28,29]. There are five *SVP* genes in *Mangifera indica*, among which *MiSVP1* and *MiSVP2* play key roles in the temperature-regulated flowering pathway [30], and *MiSVP3* and *MiSVP4* can inhibit mango flowering and participate in the regulation of mango flower formation gene network [31].

In summary, flower bud formation in plants is regulated by multiple genes acting upstream in floral induction pathways, and species-specific differences in the architecture of these upstream regulatory networks likely underlie the variation in the annual timing and distribution of flower bud formation across plant species.

Most *Camellia* species flower during winter and spring. For instance, *Camellia japonica*, the model species of the genus, typically blooms from January to March. However, *Camellia azalea* is a rare species capable of flowering year-round, making it a valuable parent for breeding camellias with continuous blooming traits. To further understand the mechanisms underlying flower bud formation and flowering regulation in *Camellia*, we compared the annual periodicity of flower bud development and leaf transcriptomes between *C. japonica* and *C. azalea*. Understanding the molecular regulatory mechanisms underlying the distinct flowering patterns of these two species is of great significance for identifying key genes associated with continuous flowering, facilitating the development of novel *Camellia* cultivars with prolonged blooming periods, and ultimately extending the ornamental viewing window of camellias.

## 2. Results

### 2.1. Annual Distribution of Flower Bud Formation Rates in C. azalea and C. japonica

Given the pronounced difference in flowering phenology between *C. azalea* and *C. japonica*, we conducted monthly surveys and statistical analyses of floral bud abundance in both species (Table 1). The survey revealed that in Hangzhou, where the average annual temperature is approximately 6 °C lower than that in the native habitat of *C. azalea* (Ehuangzhang, Yangchun, Guangdong Province, China)—the flower buds formed continuously throughout the year. Flower buds measuring less than 2 mm in length were classified as newly formed. Among the three surveyed *C. azalea* plants, the annual number of flower buds formed ranged from 250 to 265, with a consistent monthly distribution pattern. Flower bud formation peaked in April, with high levels also observed in March and May. The flower bud formation quantity declined in June, showed a slight increase in July, and then gradually decreased over the subsequent months, reaching only 1–2 buds, or none at all, in January and February. A parallel survey of three *C. japonica* plants revealed that its peak flower bud formation occurred in June, approximately two months later than in *C. azalea*, with relatively few buds formed in May and July. Annual total bud production in *C. japonica* varied among individuals, ranging from 66 to 178 buds, and this inter-individual variation was also reflected in monthly bud counts.

**Figure 1:**
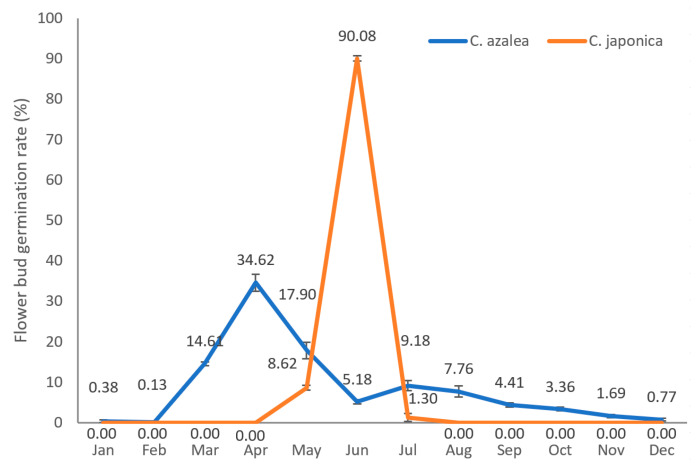
Annual germination distribution of *C. azalea* and *C. japonica* flower buds.

To minimize inter-individual variation and better compare the monthly dynamics of new flower bud formation between *C. azalea* and *C. japonica*, we generated a line graph showing the percentage of buds formed each month relative to the annual total (Figure 1). In *C. azalea*, flower bud formation occurred year-round, peaking in April (34.62%) with a secondary peak in July (9.18%). Bud production was nearly absent in January and February (<1%), consistent with its perpetual flowering habit. Notably, the flower buds formed in large numbers in April subsequently develop and bloom during the peak flowering period in July. *C. japonica* formed flower buds exclusively from May to July, with the vast majority (90.08%) initiated in June. These buds subsequently developed and bloomed from January to March of the following year, with peak flowering occurring in February.

### 2.2. Transcriptome Analysis of C. azalea and C. japonica Leaves Throughout the Year

We compared and analyzed the leaf transcriptional profiles of *C. azalea* and *C. japonica*. For *C. azalea*, transcriptome sequencing was performed and generated a total of 248.91 Gb of clean data, with Q30 values exceeding 93.7% for all samples (Supplementary Table S1). For *C. japonica*, two sequencing strategies were employed: (1) a pooled sample comprising equal amounts of RNA from 36 monthly leaf samples (three replicates per month, 12 months), which yielded 11.81 Gb of data with a Q30 of 95.2%; and (2) individual sequencing of the same 36 monthly leaf samples, which generated 58.06 Gb of clean data, with Q30 values above 95.94% per sample (Supplementary Table S2). The results of the correlation analyses and principal component analyses showed good repeatability between the three samples in the same group (Supplementary Figure S1).

By comparison with various functional databases, the unigenes of the two species were annotated separately. *C. azalea* had 35,111 unique genes annotated, with the number of annotated unigenes ranging from 16,121 to 34,247 across the databases. *C. japonica* had 85,782 unique genes annotated, with the number of annotated unigenes ranging from 26,512 to 85,228 across databases.

**Figure 2:**
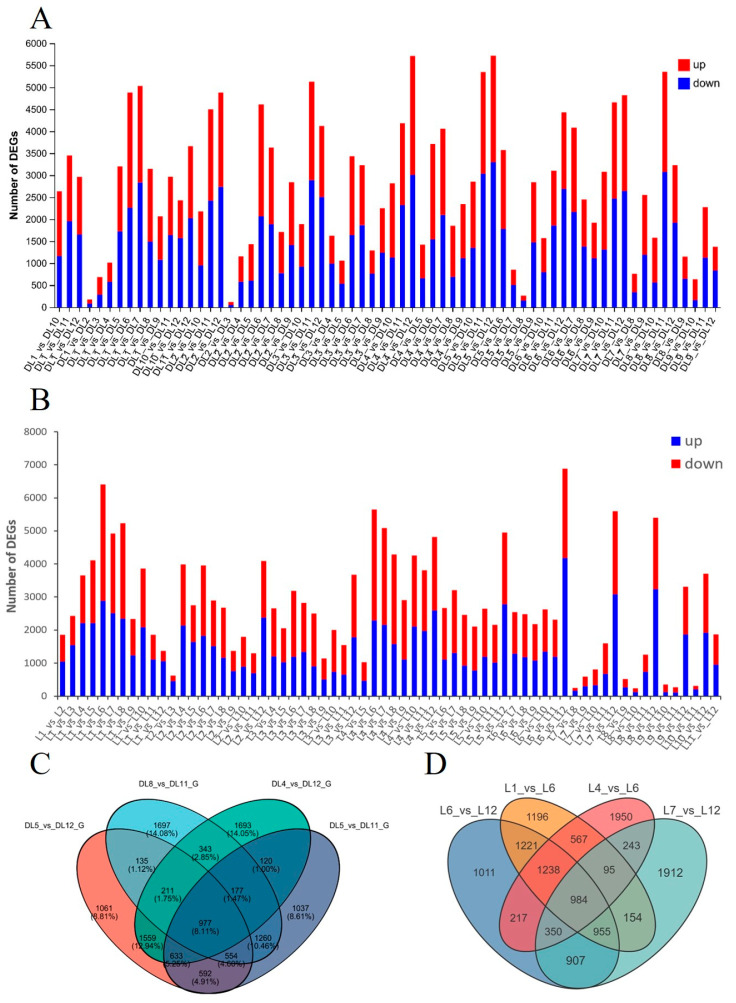
Statistical analysis of DEGs identified in *Camellia* leaves from transcriptome data between different months. (**A**) Number of DEGs between different months in *C. azalea* leaves. (**B**) Number of DEGs between different months in *C. japonica* leaves. (**C**) Venn diagram of DEGs in *C. azalea*. (**D**) Venn diagram of DEGs in *C. japonica*.

Differentially expressed genes (DEGs) were identified using DESeq2 based on raw read counts, with significance thresholds of an adjusted *p*-value < 0.05 and |log_2_(fold change)| ≥ 1. Normalized expression levels were estimated as transcripts per kilobase per million mapped reads (TPM) for *C. azalea* and fragments per kilobase per million mapped fragments (FPKM) for *C. japonica* for downstream visualization and comparison. In *C. azalea*, the transcriptome comparisons yielding the highest numbers of DEGs were DL5_vs_DL12, DL4_vs_DL12, DL8_vs_DL11, and DL5_vs_DL11, whereas the fewest DEGs were observed in DL2_vs_DL3, DL1_vs_DL2, and DL5_vs_DL8 (Figure 2A). This pattern corresponds to the periods of high flower bud formation in *C. azalea* during April, May, and August, and very low bud formation from November through February. A Venn diagram was constructed for the four pairwise comparisons with the highest numbers of differentially expressed genes (DEGs)—DL5_vs_DL12, DL4_vs_DL12, DL8_vs_DL11, and DL5_vs_DL11—showing 5722, 5713, 5354, and 5350 DEGs, respectively, with 977 DEGs shared across all four comparisons (Figure 2C). In *C. japonica*, The four groups with the highest numbers of DEGs transcriptome were L6_vs_L12, L1_vs_L6, L4_vs_L6, and L7_vs_L12, and the three groups with the lowest number of DEGs were L8_vs_L10, L7_vs_L8, and L9_vs_L11 (Figure 2B). This pattern corresponds to the peak in flower bud formation observed in June for *C. japonica*, as well as the absence of bud formation from August to April of the following year. A Venn diagram was constructed for the four pairwise comparisons with the highest numbers of DEGs—L6_vs_L12, L1_vs_L6, L4_vs_L6, and L7_vs_L12—revealing 6883, 6410, 5644, and 5600 DEGs, respectively, with 984 DEGs shared across all four comparisons (Figure 2D).

**Figure 3:**
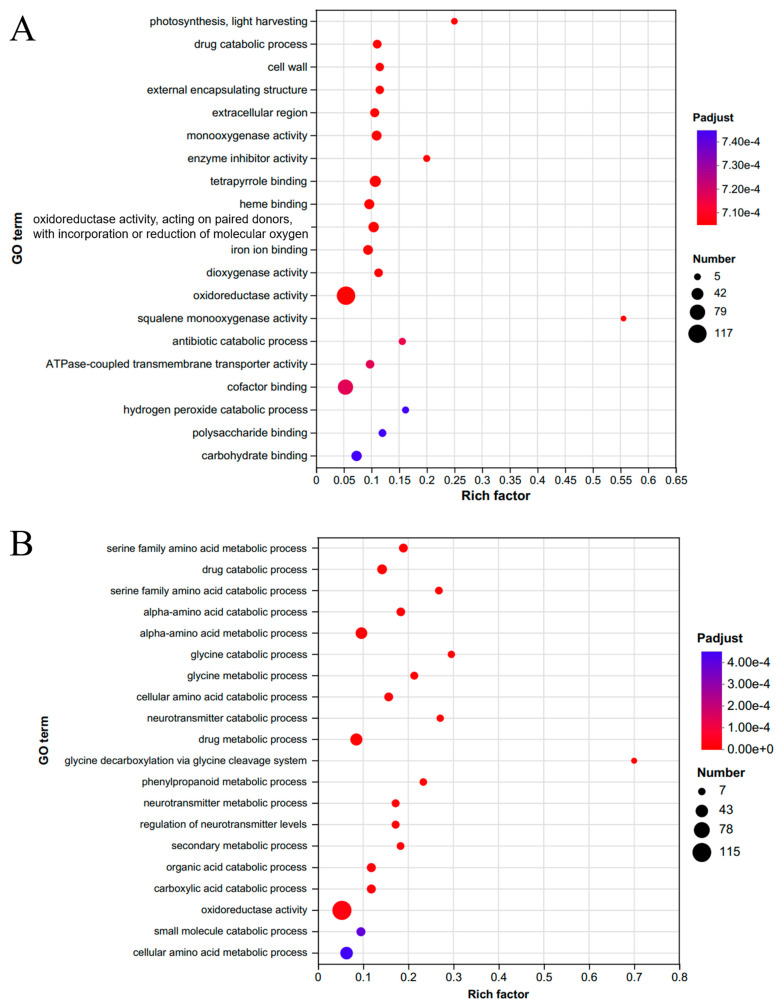
Gene ontology (GO) enrichment functional analysis for (**A**) *C. azalea* and (**B**) *C. japonica*.

Gene ontology (GO) analysis of the 977 common DEGs in *C. azalea* and the 984 common DEGs in *C. japonica* showed that the most validly enriched category in the *C. azalea* transcriptome was oxidoreductase activity, followed by cofactor binding (Figure 3A). Similarly, the most validly enriched category in the *C. japonica* transcriptome was oxidoreductase activity, followed by cellular amino acid metabolic process (Figure 3B).

Further functional analysis of these DEGs revealed a limited number of flowering-related regulators: only *ELF4* and *AP2* were identified in *C. japonica*, while *COL2* and *COL16* were detected in *C. azalea*. Therefore, we searched all known flowering-related genes to enable a targeted analysis of their differential expression levels.

### 2.3. Analysis of Flowering-Related Genes in Different Pathways Based on Transcriptome Data

Based on the transcriptome analysis of *C. azalea* and *C. japonica*, flowering-related genes were identified from the assembled transcriptomes by screening for components of six canonical floral regulatory pathways: photoperiod, thermosensitivity, GA, vernalization, autonomous, and age. These included both floral repressors (e.g., *SVP*, *FLC*-like genes) and integrators (e.g., *FT*, *SOC1*). A total of 248 and 257 flowering-related gene transcripts were obtained for *C. azalea* and *C. japonica*, respectively. Some genes have multiple transcripts, and the number of genes varies between the two species. Detailed information on the number of flowering-related genes in the transcriptomes of the two species can be found in Supplementary Table S3.

**Figure 4:**
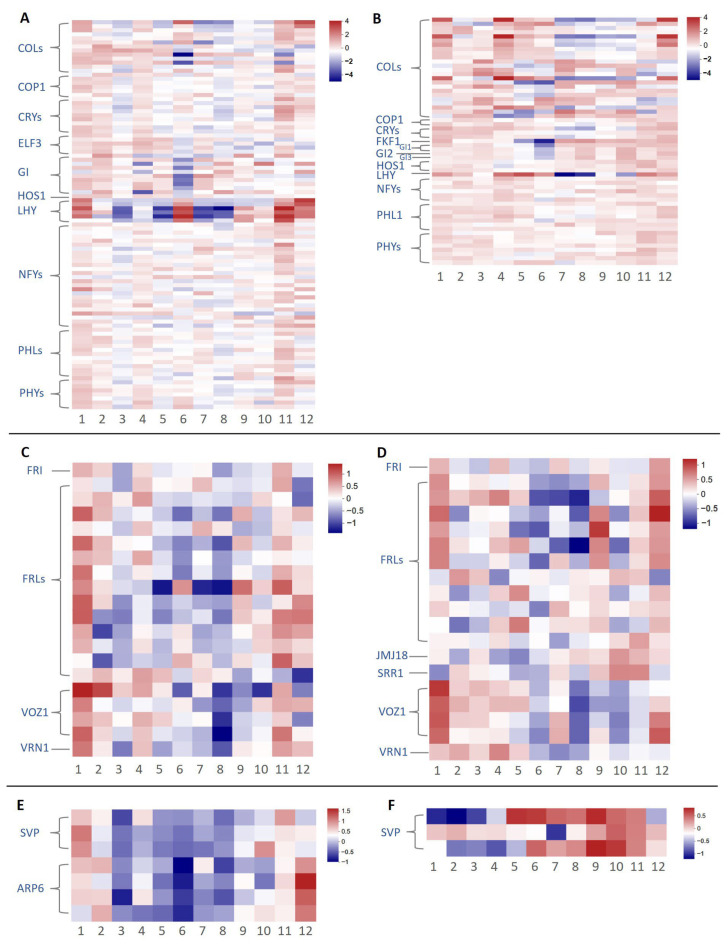
Heatmap of flowering-related genes through the photoperiodic, vernalization, and thermosensitive pathways in *C. azalea* and *C. japonica.* (**A**) Heatmap through the photoperiodic pathway of *C. azalea*. (**B**) Heatmap through the photoperiodic pathway of *C. japonica*. (**C**) Heatmap through the vernalization pathway of *C. azalea*. (**D**) Heatmap through the vernalization pathway of *C. japonica*. (**E**) Heatmap through the thermosensitive pathway of *C. azalea*. (**F**) Heatmap through the thermosensitive pathway of *C. japonica*. The numbers from negative to positive represent the changes in the log_2_-transformed relative TPM or FPKM value from low to high.

For each pathway, we excluded genes with 12-month average TPM (For *C. azalea*) or FPKM (*C. japonica*) values below 5. After filtering, 98 and 59 genes remained in the photoperiod pathway for *C. azalea* and *C. japonica*, respectively. Among these, *LHY*, *GI*, and *COLs* exhibited the most pronounced expression changes across the 12 months in both species (Figure 4A,B). Several transcripts exhibited dramatic upregulation or downregulation in June. Although the number of DEGs varied between species, most displayed clear seasonal expression patterns. Additionally, *GI2* and *FKF1* were detected only in *C. japonica*, where they showed low expression during May–June, the period of active floral bud formation, and high expression during the rest of the year.

In the vernalization pathway, 20 and 19 genes were retained for analysis in *C. azalea* and *C. japonica*, respectively, following expression filtering. Overall, vernalization-related genes showed lower expression levels compared to those in the photoperiod pathway. In *C. azalea*, these genes exhibited no clear seasonal expression pattern, with only modestly elevated expression in January and minimal variation across other months (Figure 4C). In *C. japonica*, *VOZ1* and *VRN1* were upregulated from January to May and downregulated from June to December (Figure 4D). In *C. azalea*, most genes exhibited slightly higher expression in January, but no clear patterns were observed for the other months.

In the thermosensitive pathway, seven highly expressed genes were identified in *C. azalea*, including *SVP* and *ARP6* (Figure 4E), whereas only *SVP* was detected in *C. japonica* (Figure 4F). In *C. azalea*, *SVP* was highly expressed only in January, corresponding to the observed reduction in flower bud formation during that month. In *C. japonica*, two *SVP* genes exhibited high expression from May to November, and the other from June to December. Both exhibited low expression from January to April, the period encompassing floral induction and flowering, consistent with *SVP* as a flowering repressor. *SVP* expression in *C. azalea* was more uniform across the annual cycle compared to the pronounced seasonal fluctuation in *C. japonica*.

**Figure 5:**
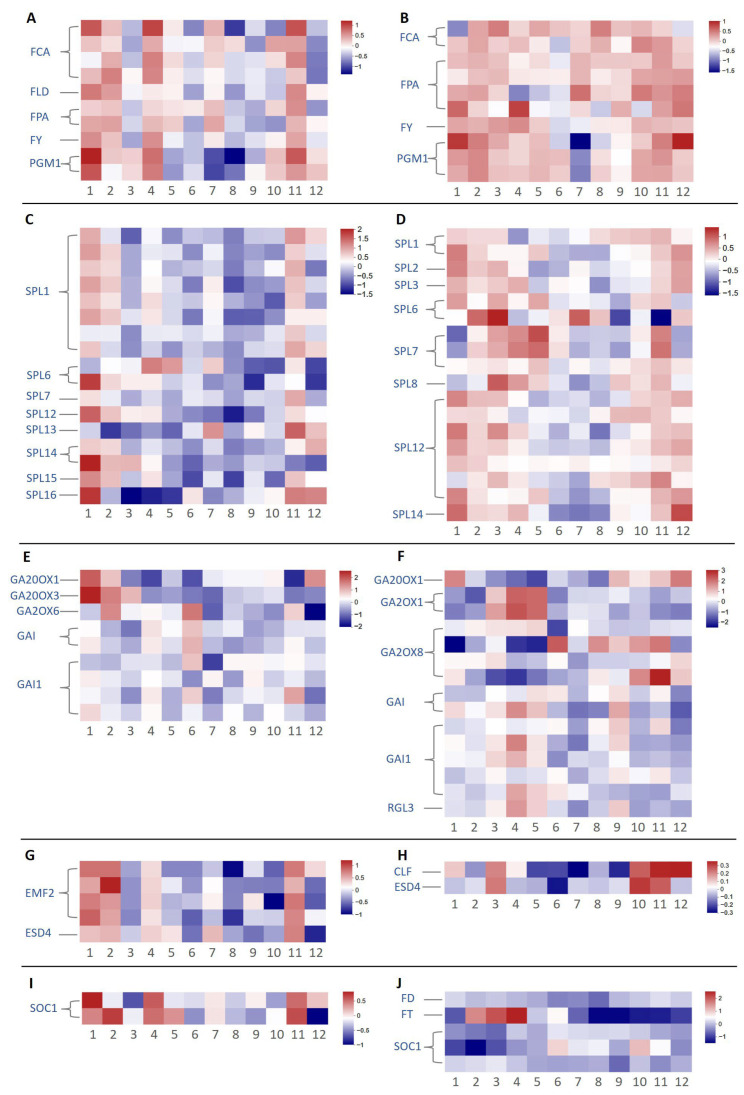
Heatmap of flowering-related genes through the autonomous, age, and GA pathways, along with flowering suppressors and flowering integrators in *C. azalea* and *C. japonica.* (**A**) Heatmap through the autonomous pathway of *C. azalea*. (**B**) Heatmap through the autonomous pathway of *C. japonica*. (**C**) Heatmap through the age pathway of *C. azalea*. (**D**) Heatmap through the age pathway of *C. japonica*. (**E**) Heatmap through the GA pathway of *C. azalea*. (**F**) Heatmap through the GA pathway of *C. japonica.* (**G**) Heatmap through flowering suppressors of *C. azalea*. (**H**) Heatmap through flowering suppressors of *C. japonica*. (**I**) Heatmap through flowering integrators of *C. azalea*. (**J**) Heatmap through flowering integrators of *C. japonica*. The numbers from negative to positive represent the changes in the log_2_-transformed relative TPM or FPKM value from low to high.

Among the genes in the autonomous pathway, *PGM1* exhibited a seasonal expression pattern over the 12 months. In *C. azalea*, *PGM1* was upregulated from October to April of the following year and downregulated from May to September (Figure 5A). In *C. japonica*, *PGM1* expression was only lower in July (Figure 5B).

All age pathway-related genes identified belonged to the *SPL* family. In *C. azalea*, *SPLs* generally exhibited low expression levels (Figure 5C), whereas in *C. japonica*, they were consistently expressed at higher levels (Figure 5D). Additionally, the number of *SPL1* transcripts was greater in *C. azalea* than in *C. japonica*. The overall higher *SPL* expression in *C. japonica* likely reflects the older developmental age of the sampled plants, as *SPL* genes are known to accumulate with plant maturity and promote the juvenile-to-adult transition.

Genes identified in GA pathway mainly fell into two categories: gibberellin oxidase genes (*GAsOXs*) and *DELLA* genes (*GAI*/*RGL*). An *RGL3* gene was also identified in *C. japonica*, but no *RGL* genes were found in *C. azalea*. In *C. azalea*, *GA20OX1, GA20OX3,* and *GA2OX6* showed variable expression, with *GA20OX1* exhibited elevated levels in December, January, and February (Figure 5E). In *C. japonica, GA20OX1, GA2OX1*, and *GA2OX8* exhibited notable variation, with *GA20OX1* showing relatively high expression in October, November, December, and January (Figure 5F). Additionally, *GA2OX1* and *GA2OX8* were exclusively detected only in *C. japonica*.

In *C. azalea*, the flowering repressors *EMF2* and *ESD4* were identified (Figure 5G), whereas *C. japonica* expressed *CLF* and *ESD4* (Figure 5H). Over the 12-month period, both species exhibited only minor fluctuations in expression: in *C. azalea*, *EMF2* and *ESD4* showed slightly elevated levels in January and February, while in *C. japonica*, *CLF* and *ESD4* peaked modestly from October to December. Overall, the magnitude of seasonal variation in these repressors was negligible in both species.

The flowering integrators identified included *FT* and *SOC1* in *C. japonica*, whereas only *SOC1* was detected in *C. azalea* (Figure 5I,J). In *C. japonica*, *FT* exhibited pronounced seasonal expression, peaking in March, April, and May—the period coinciding with floral bud formation—and remaining nearly undetectable during the rest of the year. *SOC1* expression patterns differed between species: in *C. azalea*, *SOC1* showed elevated levels in January, April, and November. In *C. japonica*, one *SOC1* paralog was highly expressed from June to November, while the other two paralogs remained consistently low throughout the year.

### 2.4. Differential Expression of Key Floral Induction Genes in Two Camellia Species

**Figure 6:**
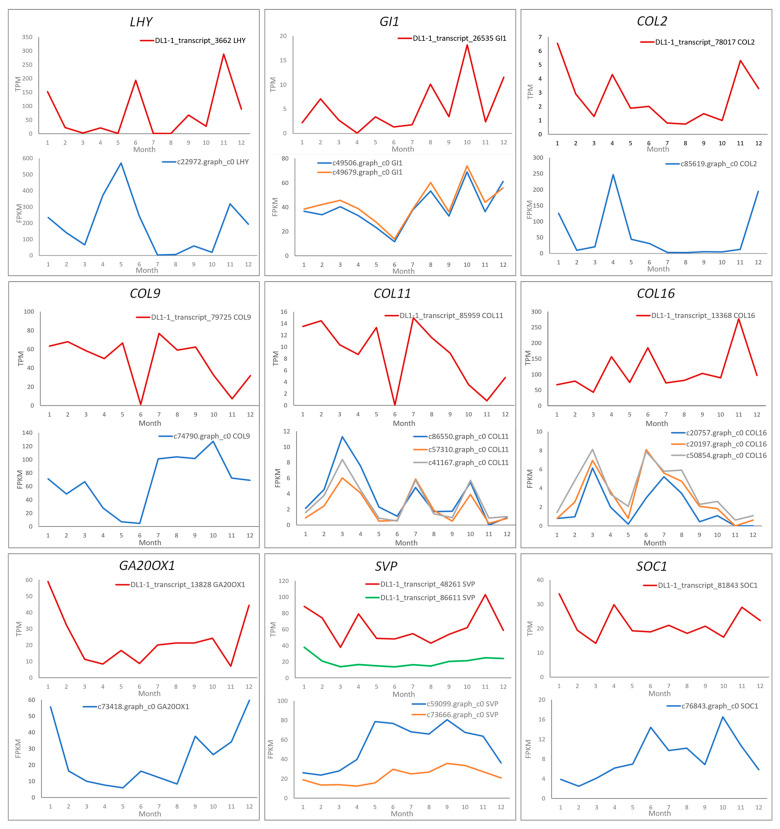
Annual expression trends of genes with differential expression across the year in *C. japonica* and *C. azalea*. DL1-1_transcript_XXX represents the transcript of *C. azalea*; cXXX.graph_cX represents the transcript of *C. japonica*.

A blast analysis was conducted on the flowering-related genes differentially expressed in *C. japonica* using the transcriptome sequences from *C. azalea*. Homologous unigenes corresponding to 20 *C. japonica* transcripts were successfully identified in *C. azalea* (Figure 6). Further comparative analysis showed that *LHY, GI1, COL2, COL9*, and *GA20OX1* exhibited similar annual expression patterns in both species. The two transcripts of the *GI1* gene in *C. japonica* exhibited consistent expression patterns.

Among the genes showing divergent expression patterns, *COL11* in *C. azalea* (DL1-1_transcript_85959) exhibited high expression levels in January, May, August, and September, whereas all three corresponding *C. japonica COL11* transcripts (c86550.graph_c0, c57310.graph_c0, and c41167.graph_c0) displayed consistently low expression throughout the year. By contrast, *COL16* maintained high expression levels throughout the year in *C. azalea*, whereas its three corresponding transcripts in *C. japonica* exhibited uniformly low expression with stable patterns over time. both species possessed two orthologous transcripts of the thermosensitive flowering regulator *SVP*, with each species exhibiting one transcript at high expression and the other at low expression. In *C. azalea*, the higher-expressing *SVP* transcript (DL1-1_transcript_48261) exhibited a high expression level only in January and November, while the lower-expressing transcript showed a relatively stable expression profile throughout the year, with a modest increase in January. In *C. japonica*, the higher-expression *SVP* transcript (c59099.graph_c0) maintained a high level from June to November, correlating with the period when new flower buds did not form. Similarly, the lower-expression *SVP* transcript in *C. japonica* also showed higher expression from June to November. The expression pattern of *C. azalea SOC1* (DL1-1_transcript_81843) was opposite to that in *C. japonica*. In *C. azalea*, *SOC1* (DL1-1_transcript_81843) peaked in January and remained low from June to October, whereas in *C. japonica*, *SOC1* (c76843.graph_c0) showed minimal expression in January and February but was highly expressed from June through November.

**Figure 7:**
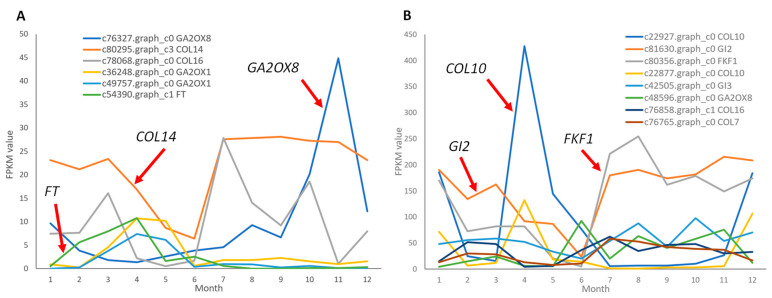
Annual expression trends of genes with differential expressions that are only expressed in *C. japonica*. (**A**) Genes with FPKM values between 5 and 50 in the highest expression month. (**B**) Genes with FPKM values above 50 in the highest expression month.

Additionally, among the 34 transcripts differentially expressed across months in *C. japonica*, 14 lacked identifiable corresponding unigenes in *C. azalea*, including *GA2OX1*, *GA2OX8*, *FT*, *COL7*, *COL14*, *FKF1*, *COL10*, and *GI2* (Figure 7). Given the substantial variation in expression levels among these genes, their annual expression dynamics were further analyzed by categorizing them into two groups based on whether their maximum FPKM value over the 12-month period exceeded or fell below 50. Among genes with FPKM values below 50 (Figure 7A), the floral integrator *FT* (c54390.graph_c1) was expressed almost exclusively from February to April, with negligible levels in all other months—its peak expression aligning precisely with the floral bud induction period in *C. japonica*. *GA2OX1* (c49757.graph_c0, c36248.graph_c0) showed an expression pattern similar to that of *FT*. *GA2OX8* (c76327.graph_c0) and *COL14* (c80295.graph_c3) showed an opposite expression trend to that of *FT*: *GA2OX8* peaked in November, while *COL14* showed a pronounced dip in expression during May and June but maintained relatively high levels throughout the rest of the year.

Among genes with FPKM values exceeding 50 (Figure 7B), *COL10* (c22927.graph_c0) exhibited pronounced monthly fluctuations, reaching its highest expression in April and showing smaller peaks in January and December. Additionally, *GI2* (c81630.graph_c0) and *FKF1* (c80356.graph_c0) displayed their lowest expression levels in May and June, with elevated expression from July to January of the following year.

### 2.5. Validation of the Key Flowering-Related Genes by qRT-PCR

**Figure 8:**
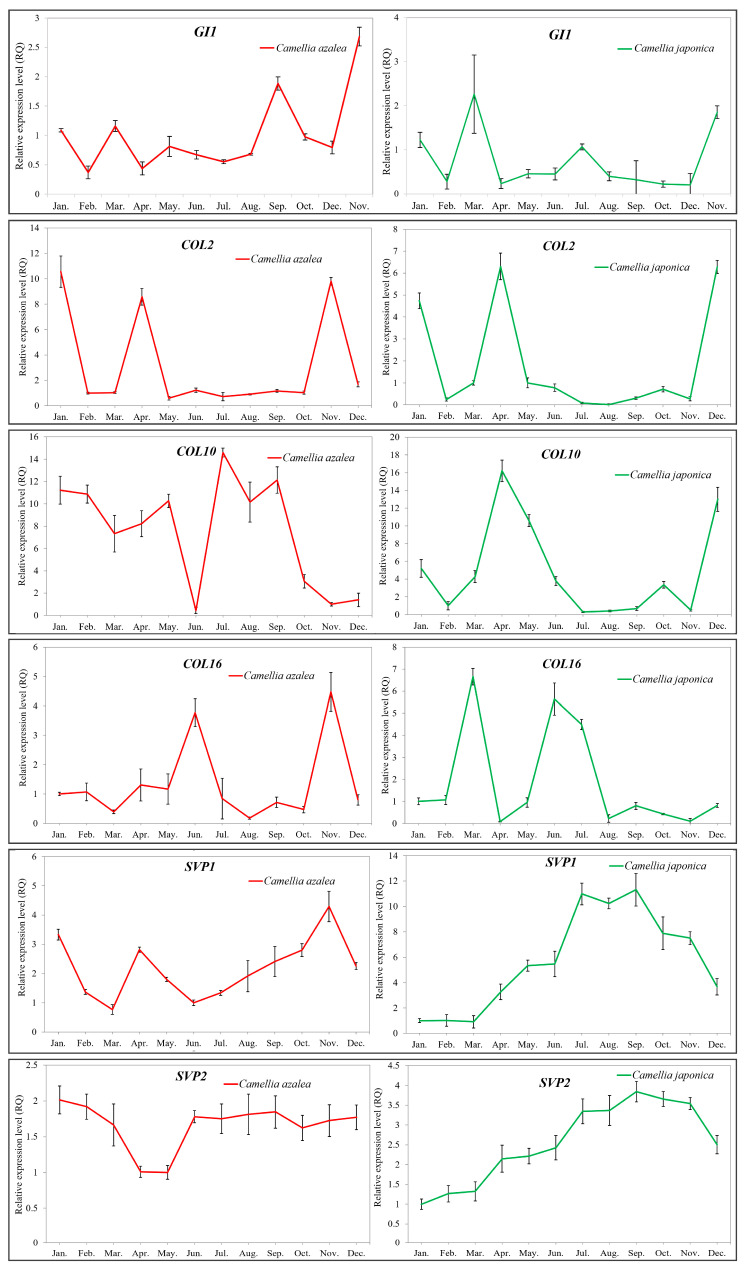
Validation of several flowering-related genes expressed in *C. azalea* and *C. japonica* by qRT-PCR analyses. *SVP1* represents the *SVP* transcript expressed at a higher level in both species shown in Figure 6, while *SVP2* represents the *SVP* transcript expressed at a lower level in both species shown in Figure 6.

To validate the reproducibility and reliability of the transcriptome data and its analysis, we performed qRT-PCR on six genes from Figure 6 (*GI1*, *COL2*, *COL10*, *COL16*, *SVP1*, *SVP2*) identified as differentially expressed in the leaves of the two *Camellia* species sampled across all months of the year. The annual expression patterns observed by qRT-PCR were generally consistent with those derived from the transcriptome data (Figure 8).

**Figure 9:**
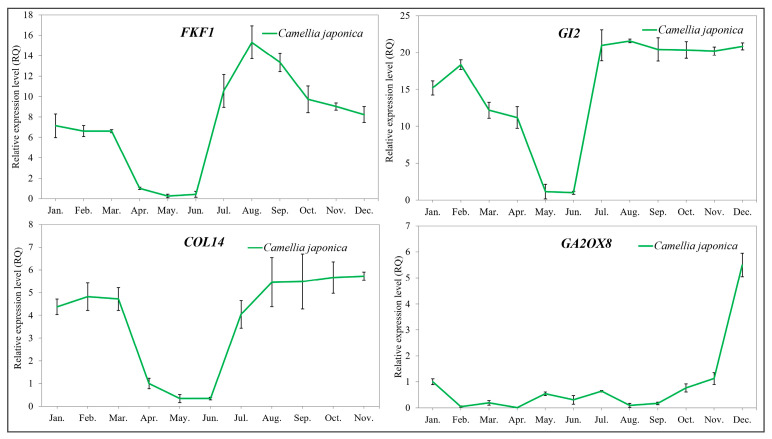
Validation of four flowering-related genes expressed only in *C. japonica* by qRT-PCR analyses. These four genes all appear in Figure 7.

Additionally, the genes *FKF1*, *GI2*, *COL14* and *GA2OX8*, shown in Figure 7, were detected exclusively in *C. japonica* via qRT-PCR, and their expression patterns align with those observed in the transcriptome (Figure 9).

## 3. Discussion

Transcriptome analysis identified 248 and 257 flowering-related transcripts in *C. azalea* and *C. japonica*, respectively. Among these genes, some were found to have multiple transcripts. These may arise from the presence of distinct gene family members—as observed for *SOC1* and *SVP*—or from alternative splicing of a single gene locus, as seen in *GI1*, *COL11*, and *COL16*. All of which are associated with the six canonical floral induction pathways. Comparative expression pattern revealed pronounced differences between the two species, particularly in genes belonging to the photoperiod and thermosensitive pathways.

In the photoperiod pathway, *GI* promotes *CO* expression by forming a complex with FKF1 that targets *CDF1* for degradation, thereby relieving CDF1-mediated repression of *CO* [7,8]. *GI* can not only promote flowering but also suppress it. For example, overexpression of *OsGI* was found to delay flowering in rice [11]. In this study, a *C. japonica*-specific member of the *GI* gene family, *GI2*, exhibited high expression during the non-floral-bud-formation period but was barely detectable during the floral bud formation period (May to June), suggesting that *GI2* may act as a repressor of floral bud formation in *C. japonica*. However, this gene was absent in *C. azalea*. This underscores its potential role as a key factor underlying the divergent flowering patterns between the two *Camellia* species. *FKF1* displayed an expression pattern similar to that of *GI2* and was detected exclusively in *C. japonica*. Thus, *FKF1* represents an important target for investigating the molecular mechanisms underlying flowering regulation. In *Arabidopsis*, *CO* and other *COL* genes constitute the *CO* gene family [10,11]. Different members of this family exhibit divergent responses to photoperiod. For example, *COL1* and *COL2* are insensitive to photoperiod [14], whereas *COL9* and *COL10* act as flowering inhibitors in *Arabidopsis* and rice, respectively [15,16]. Overexpression of the *Chrysanthemum COL16* promotes early flowering in *Arabidopsis* [17]. In our study, *C. japonica COL10* exhibited peak expression in April and maintained high expression levels in May, December, and January. No corresponding unigene was detected in *C. azalea*; Consequently, further functional validation is required to determine whether *COL10* acts as a flowering promoter or inhibitor. In contrast to its expression pattern in *C. japonica*, *COL16* was constitutively expressed at high levels throughout the year in *C. azalea*, suggesting a potential role in the latter’s year-round flowering phenotype. The *COL14* gene, detected exclusively in *C. japonica*, exhibited high expression throughout the year except during May and June—the period of floral bud formation—suggesting that it may act as a repressor of floral initiation in this species.

*SVP* encodes an important transcription factor in the thermosensitive pathway that inhibits flowering by repressing *FT* expression [28,29]. Two *SVP* genes were identified in this study that exhibited markedly divergent expression patterns across the 12 months in leaves of *C. azalea* and *C. japonica*. *SVP* expression in *C. japonica* was relatively stable within seasons, showing low levels from January to April and high levels from July to November, consistent with regulation by seasonal temperature changes. In *C. azalea*, *SVP* expression remained relatively stable and showed little response to seasonal changes. This differential regulation suggests that *SVP* may act as a key repressor of floral bud formation in *C. japonica*, while its constitutive low expression in *C. azalea* could contribute to continuous flowering.

*SOC1* is a downstream gene of *FT*. In *C. japonica*, one of the *SOC1* transcript exhibited a seasonal expression pattern, with its peak coinciding with the high-expression phase of *SVP*. In contrast, this *SOC1* expression in *C. azalea* remained stable throughout the year, whereas its seasonal upregulation in *C. japonica*—coinciding with *SVP*—suggests that this *SOC1* transcript may function atypically, potentially contributing to the repression of floral bud induction in *C. japonica*.

**Figure 10:**
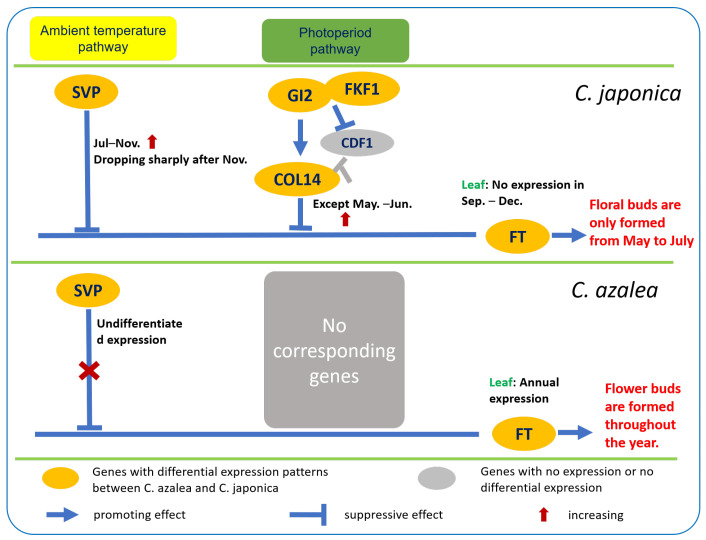
Differential molecular regulation mechanisms of floral induction in *C. azalea* and *C. japonica*.

Although *FT* transcripts were not detected in the leaf transcriptome of *C. azalea* in this study, our previous qRT-PCR analysis revealed year-round *FT* expression in its leaves, with a peak from April to May [32]—a pattern that aligns with the species’ continuous floral bud formation and coincides with its primary bud development period. In *C. japonica* leaves, *FT* expression peaked from April to June, coinciding with the floral bud induction period, and was undetectable from September to December—consistent with the absence of new floral buds during these months. This expression pattern indicates that *FT* plays a critical regulatory role in floral bud formation in both *C. azalea* and *C. japonica*. Based on transcriptome analysis, we formulated a working hypothesis and constructed a simplified regulatory network illustrating the differential control of floral induction in the two *Camellia* species, incorporating DEGs identified in the photoperiod and thermosensory pathways (Figure 10). The divergence in photoperiod pathway regulation between *C. azalea* and *C. japonica* is primarily attributed to *GI2*, *FKF1*, and *COL14*—genes absent in *C. azalea* but exhibiting almost consistent expression in *C. japonica* leaves. These three genes are highly expressed throughout most of the year, except during the floral bud formation period (May and June), suggesting that they likely act as repressors of floral initiation. The primary divergence in the thermosensitive pathway between the two species lie in *SVP* expression dynamics: *C. azalea* exhibits minimal seasonal variation, whereas *C. japonica* shows sustained high expression from July to November—corresponding to the period when floral bud formation is absent. This suggests that *SVP* acts as a repressor of floral bud formation in *C. japonica*, whereas its stable, seasonally unresponsive expression in *C. azalea* may reflect a diminished or altered regulatory role, potentially contributing to year-round flowering.

The absence of *GI2*, *FKF1*, and *COL14*, combined with the lack of pronounced seasonal variation in *SVP* expression in *C. azalea*, collectively suggest the absence of photoperiod- and thermosensitive-mediated repression of *FT* expression in this species. Consequently, *FT* is constitutively expressed throughout the year in *C. azalea*, resulting in continuous floral bud formation.

In *C. japonica*, the combined action of the photoperiod and thermosensitive pathways, marked by high expression of *GI2*, *FKF1*, *COL14*, and *SVP* from July to December, suppresses *FT* expression, consistent with the absence of floral bud formation during this period. Following a decline in *SVP* expression after November, *FT* expression in *C. japonica* increases slightly from January to March of the following year, coinciding with its flowering period. In April, the downregulation of photoperiod-associated repressors, *GI2*, *FKF1*, and *COL14*, combined with persistently low *SVP* expression, leads to a peak in *FT* expression, marking the onset of floral bud formation in *C. japonica*. By July, high expression of *GI2*, *FKF1*, *COL14*, and *SVP* resumes, repressing *FT* and thereby preventing further floral bud initiation for the remainder of the year.

Overall, we confirmed a clear difference in the annual pattern of floral bud formation between *C. azalea* and *C. japonica*: *C. azalea* is capable of forming floral buds year-round, whereas *C. japonica* initiates buds only from May to July. This phenotypic divergence is likely attributable to the absence of photoperiod- and thermosensitive-mediated repression of flowering in *C. azalea*. In general, our results suggest that the differences in the relevant upstream inhibitory pathways of floral induction in *C. azalea* and *C. japonica* may lead to the discrepancies in the annual distribution of floral bud formation between the two species. However, elucidating the specific regulatory mechanisms requires more direct validation. Overall, our results suggest that species-specific differences in the upstream repressive modules of floral induction pathways underlie the contrasting annual patterns of floral bud formation in *C. azalea* and *C. japonica*. However, elucidating the precise regulatory mechanisms will require further functional validation.

## 4. Materials and Methods

### 4.1. Plant Materials and Growth Conditions

The plant materials were selected from two *Camellia* species exhibiting distinct flowering phenotypes: *C. azalea*, a rare species that flowers year-round with a peak blooming period in summer, and *C. japonica*, the model species of the genus *Camellia*, which blooms exclusively in spring. All plants were grown under natural light conditions in the experimental field at the Research Institute of Subtropical Forestry in Hangzhou, Zhejiang, China (30°03′ N, 119°57′ E). The *C. azalea* individuals used in this study were 5-year-old field-grown seedlings, whereas the *C. japonica* plants were 10-year-old field-grown seedlings.

### 4.2. Investigation of the Annual Bud Formation Pattern

Three individual plants of *C. azalea* and three of *C. japonica* were selected for analysis. The number of floral buds measuring less than 2 mm in diameter was recorded at mid-month over a 12-month period. For each species, the monthly floral bud formation rate (%) was then calculated as the number of new floral buds observed in a given month divided by the total number of floral buds formed over the entire year, multiplied by 100. This metric enabled a comparative assessment of the seasonal distribution patterns of floral bud initiation between *C. azalea* and *C. japonica*.

### 4.3. Transcriptome Sequencing and Gene Expression Analysis of Leaves Throughout the Year

Transcriptome sequencing was performed on leaves of *C. azalea* and *C. japonica* collected at mid-month over a 12-month period, spanning from June of the current year—when tender shoots begin to lignify—through May of the following year. For each species, a cohort of continuously developing leaves was sampled monthly, with three biological replicates per month, yielding a total of 36 samples. Total RNA from these samples was extracted using the RNAprep Pure Plant Polysaccharide & Polyphenol Total RNA Extraction Kit from TIANGEN (Beijing, China). RNA samples from *C. azalea* were sent to Shanghai Majorbio Technology Co., Ltd. (Shanghai, China) for individual sequencing of all 36 samples. The RNA samples from *C. japonica* were sent to Beijing Biomarker Technologies, where the 36 samples were individually profiled for gene expression, but the transcriptome sequencing library was constructed from an equimolar pool of all 36 samples.

RNA sequencing (RNA-seq) for *C. azalea* was performed using the Illumina Novaseq 6000 platform, while both RNA-seq and expression profile sequencing for *C. japonica* were carried out using the Illumina HiSeq^TM^ 2500 platform. Transcript assembly was carried out using Trinity (https://github.com/trinityrnaseq/trinityrnaseq/wiki 30 October 2025), and the resulting full-length transcripts were treated as unigenes.

The quality of the clean data was assessed by calculating the Q20, Q30, and GC values. All acquired transcripts functionally annotated by aligning them against the following public databases: National Center for Biotechnology Information non-redundant protein sequences (NR) (https://ftp.ncbi.nlm.nih.gov/blast/db/FASTA/, 5 May 2022), Swiss-Prot (a manually annotated and reviewed protein sequence database) (http://web.expasy.org/docs/swiss-prot_guideline.html, 6 May 2022), Protein family (Pfam) (http://pfam.xfam.org/, 9 May 2022), Clusters of Orthologous Groups of proteins (KOG/COG) (http://www.ncbi.nlm.nih.gov/COG, 10 May 2022), Gene Ontology (GO) (http://www.geneontology.org, 11 May 2022), and Kyoto Encyclopedia of Genes and Genomes (KEGG) (http://www.genome.jp/kegg/, 12 May 2022). For *C. azalea*, transcript expression levels were quantified using RSEM. The transcriptome assembly was used as the reference, and expression was reported as both TPM (transcripts per million) and read counts (i.e., the number of reads mapped to each gene). For *C. japonica*, sequencing reads were aligned to the reference genome using Tophat (v2.0.13), and the gene expression levels were estimated with Cufflinks/RSE. FPKM (fragments per kilobase of transcript per million mapped reads) values were used to represent the expression abundance of individual genes. DEG expression was analyzed using five complementary methods: DESeq2, DEGseq, edgeR, Limma, and NOISeq. The significance thresholds applied were as follows: DESeq2: adjusted *p*-value < 0.05 and |log_2_(fold change)| ≥ 1; DEGseq: adjusted *p*-value < 0.001 and |log_2_(fold change)| ≥ 1; edgeR: adjusted *p*-value < 0.05 and |log_2_(fold change)| ≥ 1; Limma: adjusted *p*-value < 0.05 and |log_2_(fold change)| ≥ 1; NOISeq: probability > 0.8. Analyses of DEGs, including Venn diagram comparisons, hierarchical clustering, gene function enrichment, and expression level difference analyses, were conducted using the Majorbio Cloud Platform (https://cloud.majorbio.com/page/tools.html, 10 July 2023). The original sequencing data of *C. japonica* are deposited in NCBl Bioproject under accession No. PRNA901631, which includes annual transcriptome sequencing (SAMN31778641-SAMN31778721). The original sequencing data of *C. azalea* are deposited in the Genome Sequence Archive (GSA) under accession No. CRA031967 (https://ngdc.cncb.ac.cn/gsa, 29 October 2025).

### 4.4. Validation of the Key Genes by qRT-PCR

Total RNA extracted from *C. azalea* and *C. japonica* was reverse-transcribed into cDNA using PrimeScript™ RT Master Mix (Perfect Real Time; Takara) (Beijing, China). Subsequently, qRT-PCR detection was performed using TB Green Premix Ex Taq II (Tli RNaseH Plus, RR820, TaKaRa) (Beijing, China). The *GAPDH* gene was used as the internal control gene for normalization of relative expression levels. Amplification was carried out on a QuantStudio^®^7 Flex system (Applied Biosystems, Foster City, CA, USA) under the following conditions: pre-denaturation at 95 °C for 30 s; 40 cycles of 98 °C for 5 s and 60 °C for 30 s; followed by 95 °C for 15 s, 60 °C for 1 min, and 95 °C for 15 s. The 2^−ΔΔCT^ method was employed to determine the relative expression levels of genes across different species and various time points [33]. The primers are listed in Table 2.

## Figures and Tables

**Table 1:** Monthly flower bud germination of *C. azalea* and *C. japonica*.

	Month	Jan.	Feb.	Mar.	Apr.	May	Jun.	Jul.	Aug	Sep.	Oct.	Nov.	Dec.	Total
Ca	Plant 1	1	0	38	90	48	12	28	15	10	7	5	3	257
	Plant 2	0	1	35	80	50	15	20	22	13	9	5	1	251
	Plant 3	2	0	40	98	40	13	23	23	11	10	3	2	265
Cj	Plant 1	0	0	0	0	16	159	3	0	0	0	0	0	178
	Plant 2	0	0	0	0	6	60	0	0	0	0	0	0	66
	Plant 3	0	0	0	0	7	81	2	0	0	0	0	0	90

Ca represents *C. azalea* and Cj represents *C. japonica*.

**Table 2:** Sequences of primers for quantitative real-time polymerase chain reaction.

Species	Target Gene	Forward Primers (5′ to 3′)	Reverse Primers (5′ to 3′)
*C. azalea* and *C. japonica*	*GAPDH*	CTGTCGATGTCTCAGTGGTTGAC	TGATCTCATCATAGGAAGCCTTCTT
	*FKF1*	TTCGAGATCTTCACTGGCTATCG	TCACTGTTCCATCATCACCATGT
	*GI1*	TAGTCCTCCATTTGCGTCTTTCA	TTTTCCTCTCCTGCTGTACATCC
	*GI2*	CCCTTGCAACCTCCCATTCT	AGGCAGACCCAACACCAAAA
	*COL2*	TGACAATGACGACGAGGCTG	TCTCCACACTGAACTGGCAC
	*COL10*	GGAGGAGGAGGAGATGACCA	CGCTAGGCATTCGAGGAGTT
	*COL14*	GGGAGGTAGTGGGAGTGGAT	TCGCTACTTTTGGGACCGAC
	*COL16*	TGTGTGGGGATAGGAGGAGG	AGGAGGAGGACGAGGATGAC
	*GA2OX8*	TCGCTACTTTTGGGACCGAC	GGCTCTCTTCGACGACTACG
	*SVP1*	GACGAAGGGTGAGATAATGGAGAA	GCCCTCAAATTCAGTTCTGGAAAA
	*SVP2*	AAGACAAAGGATGACAGGGTTGA	TTCTGAAGACTGCCCTTGTTCTT

## Data Availability

The data presented in this study are available on request from the corresponding author. The data are not publicly available due to privacy restrictions.

## Supplementary Materials

The following supporting information can be downloaded at https://www.mdpi.com/article/10.3390/ijms262210854/s1.
