# Supplementary material for: Differences in the Gene Regulatory Network for Floral Induction in Two Camellia Species
Source: Int J Mol Sci. 2025 Nov 8;26(22):10854. doi: 10.3390/ijms262210854 (PMC12652118; doi:10.3390/ijms262210854)
Supplement: Supplementary file 1 [file ijms-26-10854-s001.zip › ijms-3915764-supplementary.pdf]

Supplementary Materials

Table S1. Sample information for transcriptome sequencing of *C. azalea*.

No.	Sample name	BMK ID	Notes
1	DL1_1	DL1_1	January leaf
2	DL1_2	DL1_2	January leaf
3	DL1_3	DL1_3	January leaf
4	DL2_1	DL2_1	February leaf
5	DL2_2	DL2_2	February leaf
6	DL2_3	DL2_3	February leaf
7	DL3_1	DL3_1	March leaf
8	DL3_2	DL3_2	March leaf
9	DL3_3	DL3_3	March leaf
10	DL4_1	DL4_1	April leaf
11	DL4_2	DL4_2	April leaf
12	DL4_3	DL4_3	April leaf
13	DL5_1	DL5_1	May leaf
14	DL5_2	DL5_2	May leaf
15	DL5_3	DL5_3	May leaf
16	DL6_1	DL6_1	June leaf
17	DL6_2	DL6_2	June leaf
18	DL6_3	DL6_3	June leaf
19	DL7_1	DL7_1	July leaf
20	DL7_2	DL7_2	July leaf
21	DL7_3	DL7_3	July leaf
22	DL8_1	DL8_1	August leaf
23	DL8_2	DL8_2	August leaf
24	DL8_3	DL8_3	August leaf
25	DL9_1	DL9_1	September leaf
26	DL9_2	DL9_2	September leaf
27	DL9_3	DL9_3	September leaf
28	DL10_1	DL10_1	October leaf
29	DL10_2	DL10_2	October leaf
30	DL10_3	DL10_3	October leaf
31	DL11_1	DL11_1	November leaf

No.	Sample name	BMK ID	Notes
32	DL11_2	DL11_2	November leaf
33	DL11_3	DL11_3	November leaf
34	DL12_1	DL12_1	December leaf
35	DL12_2	DL12_2	December leaf
36	DL12_3	DL12_3	December leaf

Table S2. Sample information for transcriptome sequencing of *C. japonica.*

No.	Sample name	BMK ID	Notes
1	L1_1	E01	January leaf
2	L1_2	E05	January leaf
3	L1_3	E06	January leaf
4	L2_1	E02	February leaf
5	L2_2	E07	February leaf
6	L2_3	E08	February leaf
7	L3_1	E09	March leaf
8	L3_2	E10	March leaf
9	L3_3	E11	March leaf
10	L4_1	E12	April leaf
11	L4_2	E13	April leaf
12	L4_3	E14	April leaf
13	L5_1	E15	May leaf
14	L5_2	E16	May leaf
15	L5_3	E17	May leaf
16	L6_1	E18	June leaf
17	L6_2	E19	June leaf
18	L6_3	E20	June leaf
19	L7_1	E21	July leaf
20	L7_2	E03	July leaf
21	L7_3	E22	July leaf
22	L8_1	E23	August leaf
23	L8_2	E24	August leaf
24	L8_3	E25	August leaf
25	L9_1	E26	September leaf

No.	Sample name	BMK ID	Notes
26	L9_2	E27	September leaf
27	L9_3	E28	September leaf
28	L10_1	E29	October leaf
29	L10_2	E30	October leaf
30	L10_3	E31	October leaf
31	L11_1	E32	November leaf
32	L11_2	E33	November leaf
33	L11_3	E34	November leaf
34	L12_1	E35	December leaf
35	L12_2	E36	December leaf
36	L12_3	E37	December leaf

Table S3. The flowering regulatory genes in the transcriptome of two species.

Gene	Ca	Cj	Gene	Ca	Cj	Gene	Ca	Cj
Photoperiod pathway			Thermosensitive pathway			GA pathway		
GI	15	8	*SVP*	3	5	*GAI*	7	12
COLs	21	41	*ARP6*	5	0	*GAI1*	5	8
PHYA	3	6	Vernalization pathway			*RGL3*	0	1
PHYB	2	6	*FRI*	18	22	*D8*	0	1
PHYC	3	1	*VOZ1*	4	4	*GA20OXs*	4	1
PHYE	4	1	*VRN1*	1	2	*GA2OXs*	1	19
CRY1	5	3	*SRR1*	1	1	Flowering suppressors		
CRY2	3	2	*TEM1*	0	2	*EMF1*	1	3
CRYD	3	2	*JMJ18*	0	1	*EMF2*	5	0
LHY	5	2	Autonomous pathway			*EMF2B*	2	0
CCA1	1	0	*FCA*	7	4	*CLF*	1	4
ELF3	7	0	*FPA*	10	4	*ESD4*	1	2
COP1	8	4	*FLD*	3	0	Flowering integrators		
HOS1	3	4	*FY*	1	2	*SOC1*	2	7
PHLs	15	10	*PGM1*	3	14	*FD*	2	1
NFYs	34	16	Age pathway			*FT*	0	1
FKF1	0	1	*SPLs*	29	29	Count	248	257

Ca represents *C. azalea*, and Cj represents *C. japonica*.

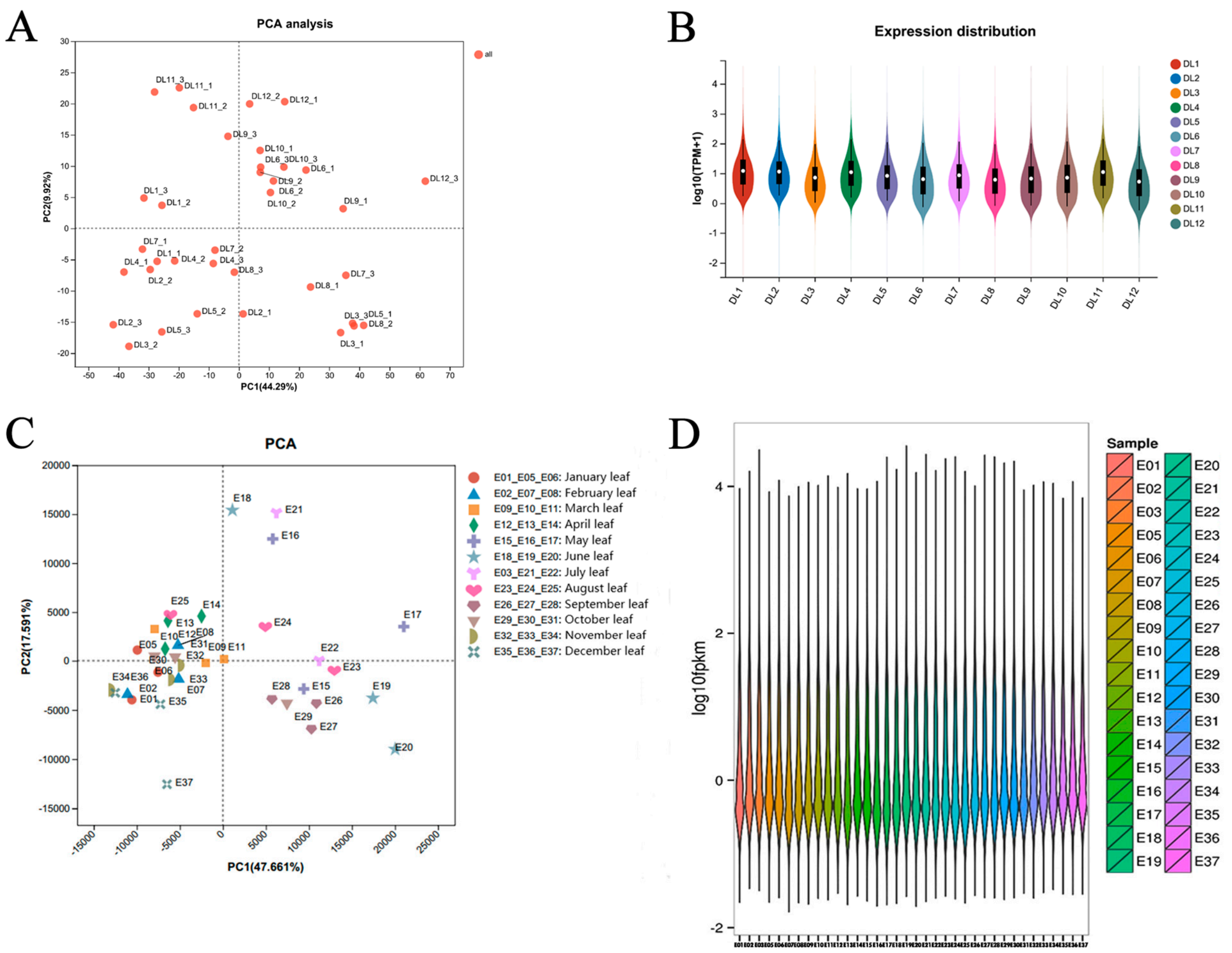

Figure S1. The correlation analysis and principal component analysis between the samples. (A) PCA analysis between the samples of *C. azalea*. (B) The principal component analysis between the samples of *C. azalea*. (C) PCA analysis between the samples of *C. japonica*. (D) The principal component analysis between the samples of *C. japonica*.
